# Universal health coverage in fragile and conflict-affected States: insights from Somalia

**DOI:** 10.1186/s12939-025-02486-3

**Published:** 2025-05-07

**Authors:** Zlatko Nikoloski, Mubarik M. Mohamoud, Elias Mossialos

**Affiliations:** 1 LSE Health, LSE, London, UK; 2 Community Nexus Consulting, Hargeisa, Somalia

**Keywords:** UHC index, Somalia, Equity analysis, Access to healthcare

## Abstract

**Introduction:**

Achieving Sustainable Development Goal (SDG) 3, which focuses on health, and more specifically SDG 3.8—universal health coverage (UHC)—by 2030 remains a critical objective for all nations. This paper presents an updated evaluation of Somalia’s progress toward UHC. Additionally, by identifying key barriers to achieving UHC in Somalia, this study offers insights that may be valuable for other conflict-affected and post-conflict countries striving to improve healthcare access and financial protection.

**Methodology:**

To assess Somalia’s progress at various levels, we developed a UHC index incorporating access to essential healthcare services and financial risk protection. Furthermore, we employed standard analytical methods, including equity analysis and logit modelling, to examine the key factors influencing both healthcare access and the financial burden associated with seeking medical care.

**Results:**

With an overall UHC index score of 33.5, Somalia ranks lowest among the countries previously analysed. While there is some regional variation in UHC scores, these differences are not as pronounced as the disparities in poverty rates, resulting in a weak correlation between regional socio-economic development (measured by poverty levels) and overall UHC performance. Equity analysis highlights that socio-economic status, educational attainment, and, to a lesser extent, healthcare infrastructure significantly contribute to disparities in access to essential health services, favouring wealthier populations. Additionally, financial risk protection analysis indicates that the most economically disadvantaged groups are at a higher risk of experiencing catastrophic healthcare expenditures.

**Implications:**

Given Somalia’s ongoing security challenges, achieving SDG 3.8 by 2030 remains a formidable task. However, targeted interventions that address key determinants—such as household income, education levels, and healthcare infrastructure—could help improve access to essential health services and reduce financial barriers to care.

**Supplementary Information:**

The online version contains supplementary material available at 10.1186/s12939-025-02486-3.

## Introduction

One of the primary objectives of Sustainable Development Goal (SDG) 3 is achieving universal health coverage (UHC) (SDG 3.8), ensuring that individuals have access to essential healthcare services while being protected from financial hardship when seeking medical care [1]. In other words, UHC asserts that all individuals should have access to the healthcare they need without experiencing significant financial hardship. Various indicators have been used to measure progress toward this goal across low-, middle-, and high-income countries [2–4]. However, there has been limited research on conflict-affected and fragile states. More specifically, only 7 of the 35 conflict-affected and fragile states have been included in existing efforts to calculate UHC performance metrics [3]. The World Bank defines such settings as those characterized by weak institutional and policy environments, the presence of a UN peacekeeping operation, or large-scale refugee flows—conditions that reflect major political or security crises [5]. The limited research in these contexts is largely attributable to data scarcity, driven by factors such as inadequate funding, the absence of up-to-date census data, and broader constraints on data availability [6]. Calculating a UHC index in the context conflict-affected and fragile states—both at national and subnational levels—is crucial, given the significant challenges these nations face in meeting SDG 3.8 and broader health-related goals.

Somalia, a low-income country, has been particularly affected by prolonged conflict. Since gaining independence in the 1960s, the nation has endured ongoing political instability and violence [7]. Decades of internal conflict have resulted in the displacement of approximately three million people, many of whom reside in camps for internally displaced persons [8]. Furthermore, Somalia ranks 23rd on the Armed Conflict Location and Event Data (ACLED) index, with security conditions described as persistently volatile [7]. The presence of armed groups such as Al-Shabaab continues to pose a major threat, and 2022 was reported as the deadliest year since at least 2018, with over 6,500 fatalities—nearly double the number recorded in 2021 [7].

### Somalia’s healthcare system

The broader security situation has significant implications for the organization and delivery of healthcare services across the country. Somalia’s healthcare system remains highly fragmented, largely due to prolonged political instability. The collapse of the central government led to the formation of three separate healthcare administrations—Somaliland, Puntland, and South-Central Somalia—each with its own Ministry of Health [9]. Somaliland, located in the northwest, declared independence in 1991 but is not internationally recognized. This region has maintained relative stability and demonstrates better health outcomes compared to the other two [10]. Puntland, in the northeast, operates under a semi-autonomous government, while South-Central Somalia, the most insecure and rural region, continues to grapple with instability, particularly due to the presence of Al-Shabaab, an armed group affiliated with Al-Qaeda [9].

Healthcare services in Somalia are provided through both public and private institutions, with private providers playing a dominant role [11]. The public sector consists of both, primary and secondary healthcare infrastructure [12]. However, private healthcare facilities tend to offer better quality services, advanced diagnostic capabilities, and more experienced staff. As a result, individuals with chronic or severe medical conditions, such as cancer, often prefer private healthcare options.

The governance of Somalia’s healthcare system is shared among the federal government, federal member states, and regional administrations. Somaliland, Puntland, and South-Central Somalia collectively form 17 federal member states, each with its own healthcare management approach. While the federal government oversees healthcare regulations through the Ministry of Health, regional authorities have taken on a significant role in decision-making. Somaliland, for example, has been working on a strategic plan aimed at achieving Universal Health Coverage (UHC) [13].

A major challenge facing Somalia’s healthcare sector is the shortage of medical professionals, exacerbated by ongoing conflict and insecurity. Many healthcare workers have left the country or moved from rural areas to urban centres, leading to an uneven distribution of medical personnel [12]. The public sector struggles to retain doctors and nurses due to low wages, prompting many to seek employment in both public and private facilities [9]. Somalia has one of the lowest physician-to-population ratios, with only 0.023 doctors and 0.11 nurses per 1,000 people, significantly lower than neighbouring Ethiopia [14]. Additionally, disruptions in medical education from 1991 to 2012 have resulted in many healthcare workers receiving inadequate training [15, 16].

Beyond workforce shortages, the healthcare system faces a critical lack of essential medicines and medical supplies. Many facilities struggle with basic necessities such as clean water and electricity, while advanced medical technologies for procedures like cancer treatment, surgery, and dialysis are scarce [16, 17]. The country also lacks a centralized regulatory body to oversee drug quality and imports, which worsens these supply-chain issues [16]. During the COVID-19 pandemic, Somalia faced severe shortages due to its reliance on imported medications [8].

A key factor hindering healthcare development is the lack of sustainable government funding [9]. With only 5% of healthcare expenditures covered by the government, most financial support comes from external donors, including international organizations and humanitarian agencies [12]. This reliance on foreign aid makes long-term healthcare improvements difficult to sustain [16].

The systemic challenges in Somalia’s healthcare system impact both the provision of UHC and financial risk protection. This study aims to provide an in-depth assessment of the country’s progress toward UHC while identifying key barriers that may also be relevant to other post-conflict nations. However, due to security concerns, data collection was limited in certain regions, which is acknowledged as a constraint in this research.

## Methods

### UHC index

To evaluate Somalia’s progress toward Universal Health Coverage (UHC), we modified Wagstaff and Neelsen’s index, a widely used measure of service accessibility and financial protection [3, 4]. This index assesses whether individuals can obtain necessary healthcare services regardless of their financial status and whether they are protected from excessive out-of-pocket expenses [3, 4]. The index takes values between 0 and 100; higher values indicate better UHC performance [4]. Although several methodologies exist for constructing a UHC index [2], we selected this approach for three main reasons: (1) it has been applied in 111 countries worldwide [3], (2) it is used by the World Bank for UHC progress tracking [18], and (3) it integrates the advantages of both, a composite index and a dashboard-style approach [4].

We tailored this index for both national and regional assessments. The indicators and their definitions, listed in Appendix Table A1, include four of Wagstaff and Neelsen’s key measures: four antenatal care visits, full immunization, professionally assisted childbirth, and care-seeking for common childhood illnesses. Additionally, we included their financial risk protection indicator, which measures catastrophic healthcare expenditure [3]. However, we omitted cervical and breast cancer screening from our analysis due to the unavailability of sub-national data.

Most indicators were measured in terms of population coverage, except for financial protection and hospital admissions. The financial protection indicator was calculated as 100 minus the percentage of households incurring catastrophic healthcare costs, defined as expenditures exceeding 10% (or 25% in sensitivity analyses) of total household consumption. To standardize inpatient admissions, we used a measure proposed by WHO. It entails 0.1 admissions per capita, amounting to 9.03% of the population that have been admitted to hospital in the past year [19].

The final index was derived as the geometric mean of two equally weighted dimensions: service coverage and financial protection. Service coverage was further divided into prevention (25%) and treatment (75%), reflecting relative spending patterns in these areas [3]. While the UHC index places emphasis on preventive healthcare services, its weighting is derived from the costs incurred at the primary and secondary care levels [3]. The prevention domain consisted of two equally weighted indicators—four antenatal care visits and full immunization—while the treatment domain included four indicators: skilled birth attendance, care-seeking for acute respiratory infections and diarrhoea, and inpatient admissions. The index was computed at both national and regional levels and compared with existing poverty headcount data [20].

### Statistical analysis

In addition to constructing the UHC index, we performed statistical analyses to further examine disparities in service coverage and financial protection.

To assess equity in access to healthcare, we applied the concentration index (CI) and decomposition analysis. There are several reasons for employing the concentration index (CI) in this analysis. First, the CI has been consistently applied in the assessment of health inequities, particularly in studies utilizing household-level data. Second, alternative measures—such as the Theil index or Gini coefficient—are primarily designed to capture economic rather than health-related disparities [21]. The CI helped measure disparities in healthcare utilization at both national and regional levels, while the decomposition analysis identified key factors contributing to these inequalities, such as education and socioeconomic status [21]. Appendix 2 provides a detailed account of variables used in the decomposition analysis. Decomposition analysis elicited some of the barriers associated with seeking preventative and treatment healthcare services. However, it is important to note the CI’s limitations, particularly the “bounds issue,” which affects comparisons across countries and time periods. For example, when two regions exhibit different mean rates of service utilization, identical concentration index (CI) values may nonetheless reflect differing degrees of inequality in access. This occurs because the mean of the distribution constrains the range of possible CI values. A limited number of studies have addressed this methodological limitation [22]. Since our analysis focused solely on Somalia at one point in time, this limitation had minimal impact [23, 24].

Given the limitations of CI in analysing financial protection, we used logistic regression models to identify the primary factors influencing catastrophic healthcare expenditure [25]. Given the binary nature of the dependent variable, a logistic regression model was employed, as it is more appropriate than alternative specifications and facilitates straightforward interpretation of the results. These models controlled for key household characteristics, as detailed in Appendix 3.

### Data sources

Our study relied on two primary datasets: the Somali Health and Demographic Survey (SHDS) and the Somali Integrated Household Budget Survey (SIHBS-22).

The SHDS provides national estimates on maternal and child health indicators and covers all 17 pre-war administrative regions. It employs a stratified multi-stage cluster sampling design, with separate sampling strategies for urban, rural, and nomadic populations. Due to security concerns, data collection was not possible in certain regions, including parts of Lower Shabelle, Middle Juba, and Bay [10].

The SIHBS-22 survey, designed to capture socioeconomic indicators, covered a sample of 7,212 households across urban, rural, and nomadic communities. The survey followed a stratified multi-stage cluster sampling approach, with probability-proportional selection methods for primary and secondary sampling units. Data collection involved direct interviews with household heads or their spouses, ensuring a high national response rate of 96% [26].

## Results

Table 1 presents the main UHC index findings. Overall, the UHC index in Somalia is low, amounting to 33.5 on the 0 to 100 scale. Second, we found limited heterogeneity in the UHC index across regions. More specifically, most subnational UHC indexes varied by +/- 5 index points from the national average. It could be argued that this lack of variation in the UHC index is the reason for the low correlation between the overall poverty rate at sub-national level and the UHC index (see Appendix Figure A1). While, as expected, we found a negative correlation between the UHC index and the poverty rate at the subnational level, the magnitude of the correlation coefficient was low (-0.2). Table 1 also reveals the coverage of each specific indicator, suggesting that the coverage of indicators is higher in the more affluent parts of the country (e.g., Awdal).

**Table 1:** Somalia: UHC index and coverage of selected interventions (as %), National and subnational analysis

	Antenatal care coverage	Full immunization	Medical assistance at delivery	Diarrhea treatment	ARI treatment	Inpatient admissions index	Catastrophic healthcare expenditure at 25%	Catastrophic healthcare expenditure at 10%	UHC
Overall	8.4	17.2	31.4	46.5	34.1	3.54	0.5	2.6	33.5
Awdal	25.8	13.0	53.7	74.3	36.1	1.55	0.4	3.0	32.3
Bakool	4.3	24.6	29.2	50.6	17.6	2.88	0.0	0.5	30.7
Banadir	5.7	17.2	38.0	36.1	31.6	2.66	0.0	2.1	30.8
Bari	4.3	24.6	16.0	37.5	33.3	4.32	0.9	4.1	32.0
Bay	15.1	21.5	31.5	47.8	45.5	5.32	0.0	0.6	39.2
Galgaduud	7.2	21.7	18.6	36.4	27.5	3.88	0.5	1.8	32.3
Gedo	1.6	21.9	10.7	28.2	6.4	17.39	0.0	0.0	34.2
Hiraan	4.4	20.4	33.9	47.6	22.9	2.44	0.6	1.1	30.0
Lower Juba	3.9	18.9	28.9	64.2	35.4	1.99	0.0	0.8	29.6
Lower Shab						3.43	0.0	0.7	
Waqooyi Ga	16.2	4.4	32.3	61.9	33.0	2.88	0.4	4.3	31.1
Middle Sha	6.7	13.1	43.3	48.8	46.7	1.88	0.0	0.0	30.5
Mudug	3.2	28.9	15.4	27.9	41.9	2.55	2.0	8.8	27.8
Nugaal	5.5	34.0	15.7	34.7	34.8	1.33	0.0	1.9	26.8
Sanaag	10.7	11.4	37.1	57.4	40.3	1.33	1.9	7.9	27.8
Sool	12.8	5.6	42.2	48.4	60.1	0.78	0.9	3.8	25.3
Togdheer	19.7	5.7	50.6	56.3	50.3	5.65	1.1	2.4	38.5

**Fig. 1:**
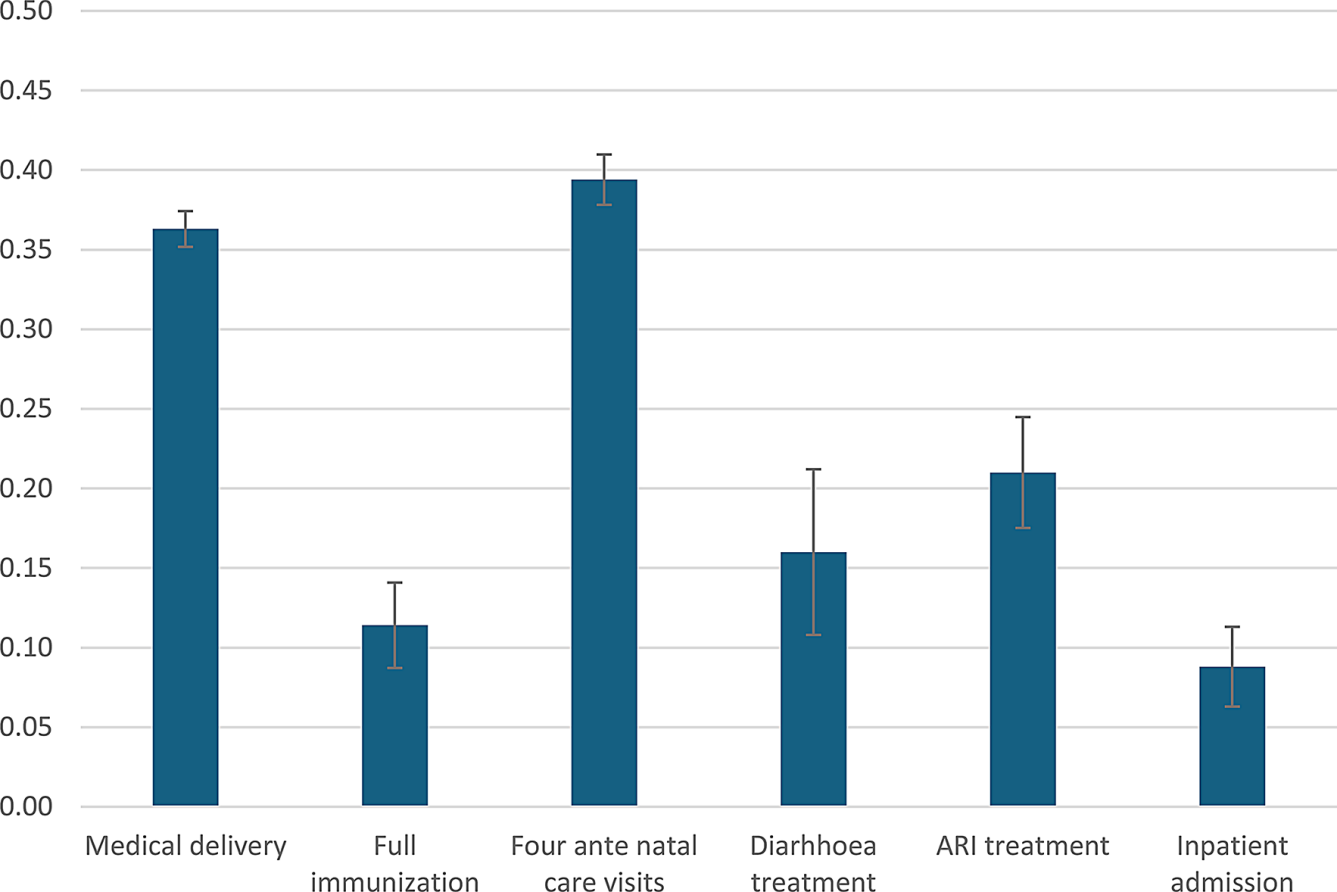
Somalia: Concentration Index (CI) (value and 95% confidence interval) for selected prevention and treatment interventions. Source SDHS, 2020 and SIHBS, 2022 and author’s calculations

While overall coverage of various indicators is revealing, it may mask access inequity. To address this issue, we conducted concentration index (CI) analysis (Fig. 1). The results indicate a significant pro-rich inequity in access to all the interventions included in the UHC index. More specifically, the magnitude of the CI was highest for having received at least four antenatal care visits (0.39) and for medical assistance during delivery (0.36), and was smallest, though still positive, for inpatient admissions, indicating pro-rich access inequity.

We coupled the CI analysis with a CI decomposition analysis to examine the extent to which different factors (e.g., socio-economic status, age) impact the observed pro-rich inequity reported above. As all coverage indicators reported pro-rich inequity in access, we conducted decomposition analysis on all of them (see results in Appendix Table A2). Two principal results arise from this analysis. First, socio-economic status (captured by the wealth index) makes the biggest contribution to observed socio-economic inequity in access to the selected interventions. Second, educational attainment status plays a significant role in accessing certain healthcare services. This was particularly pronounced in the case of professionally-assisted childbirth at a healthcare facility and full immunization of children under 5 years. In some instances, the urbanicity variable also increased inequity in access to certain services, which is potentially explained by the availability of some healthcare services in urban areas only.

Table 2 presents the findings from a simple model of CHE correlates when considering the 10% threshold. This model presents a number of findings. First, we found that the probability of experiencing CHE decreases with household socio-economic status. More specifically, households in the fourth quintile are 0.37 times less likely to experience CHE relative to households in the bottom quintile. Similarly, households in the top quintile of the socio-economic distribution are 0.4 times less likely to experience CHE when using healthcare, relative to households in the bottom quintile. Similar findings were observed at the 25% threshold; however, in this instance, the socio-economic status variable was insignificant (the full set of results are reported in the appendix).

**Table 2:** Logistic regression, determinants of CHE at 10% cut-off

che_10	Coef.		St.Err.	t-value		*p*-value	[95 % Conf		Interval]	Sig
Age of household head	1.000		0.001	-0.45		0.650	0.998		1.001	
Household head is female	1.207		0.251	0.90		0.366	0.803		1.815	
Household head went to school	1.166		0.323	0.55		0.580	0.678		2.005	
Urban	1.000		.	.		.	.		.	
Rural	0.718		0.180	-1.32		0.186	0.440		1.173	
Nomadic	1.109		0.352	0.33		0.745	0.595		2.067	
1 quintile	1.000		.	.		.	.		.	
2.quintile	1.015		0.312	0.05		0.961	0.556		1.853	
3.quintile	0.726		0.277	-0.84		0.401	0.343		1.534	
4.quintile	0.377		0.168	-2.19		0.028	0.158		0.901	**
5.quintile	0.400		0.200	-1.83		0.067	0.150		1.065	*
Constant	0.045		0.020	-6.80		0.000	0.018		0.109	***
Mean dependent var		0.025			SD dependent var			0.157		
Pseudo r-squared		0.090			Number of obs			6359.000		
Chi-square		91.396			Prob > chi2			0.000		
Akaike crit. (AIC)		500851.153			Bayesian crit. (BIC)			501013.336		

*** *p* < 0.01, ** *p* < 0.05, * *p* < 0.1

*The models also include regional fixed effects*

## Discussion

This study aimed to provide a comprehensive assessment of the country’s progress toward achieving Universal Health Coverage (UHC), while identifying critical barriers that may hold relevance for other post-conflict settings. To the best of our knowledge, this is a first study to undertake such an analysis. Furthermore, this study applies the established UHC measurement methodology to the Somali context—at both national and subnational levels—drawing on the most recent and only available Demographic and Health Survey, as well as the Household Budget Survey. A number of findings stem from this analysis. First, the UHC index is low at both national and regional levels. While some index heterogeneity exists across regions, variation at the subnational level is not as large as the variation in poverty rates, leading to a somewhat low level of correlation between regional level socio-economic development (proxied by poverty) and the overall UHC index. The results of the equity analysis indicate that socio-economic status and educational attainment (and, to some extent, availability of healthcare infrastructure) contribute to pro-rich inequity in access to selected basic healthcare interventions. Finally, the results of the financial risk protection analysis suggest that those who are most socio-economically deprived are most likely to experience catastrophic healthcare expenditure.

With an overall value of 33.5, our results place Somalia last in the list of 120 countries included in previous analyses [3]. Some of the countries in Somalia’s immediate neighborhood fare only marginally better (Ethiopia is penultimate in the list). Moreover, given that we excluded two cancer screening indicators from our analysis, owing to the lack of availability of recent sub-national data, Somalia’s overall UHC index might be lower still. The low UHC index reflects several systemic healthcare challenges including, among others, fragmented governance structures [11, 12], shortages of medical professionals [12], limited availability of essential medicines [16, 17], and inadequate financing of the public healthcare system [9].

Access to preventative and treatment services tends to favour the richer part of the population. In that respect, Somalia is similar to the wider Middle East and North Africa (MENA)/Horn of Africa region, where access to many basic healthcare services favors the more socio-economically privileged [27]. In the context of Somalia, it has recently been shown that access to some of key interventions relevant to child and maternal healthcare is low, heightening the risk of child and maternal mortality [28]. More importantly, despite an increase in the use of healthcare between 2006 and 2019, access to key interventions that would improve overall child and maternal health remains low and heavily skewed toward those who are socio-economically privileged [29].

Several factors contribute to the underutilization of healthcare services in low-income and fragile states. Although maternal healthcare services are generally provided free of charge, additional barriers may prevent women from seeking care [30]. These obstacles include transportation costs and expenses associated with having an accompanying person [30]. Beyond financial limitations, perceptions of healthcare quality also influence individuals’ willingness to access medical services [31].

Cultural and societal norms further restrict women’s access to maternal healthcare. In some cases, women are unable to seek prenatal care without a male chaperone [32]. Additionally, when emergency medical procedures such as caesarean sections are required, physicians often need the male head of household’s consent before proceeding. Delays in obtaining this consent, often due to misconceptions about medical interventions during childbirth, can put women’s health at greater risk [33]. Another major factor affecting maternal health outcomes is the prevalence of female genital mutilation (FGM) in certain regions, which increases complications during labor and delivery [32].

Furthermore, decomposition analysis supports findings from previous research indicating that maternal education levels and household socioeconomic status significantly contribute to disparities in access to maternal healthcare services, with wealthier households benefiting disproportionately [34–36].

Finally, the results of the financial risk analysis suggest that the extent of CHE in Somalia is low and, more importantly, that the poor are more likely to experience financial catastrophe when seeking healthcare. These findings are consistent with previous evidence from other low and lower middle-income countries [20, 37–39]. The low extent of CHE in Somalia is typical of a low-income country, where over three-quarters of the typical household budget is spent on food. In other words, the very low percentage of households with catastrophic healthcare expenditure, as is the case of Somalia, is indicative of system failure; i.e., the poor simply do not seek healthcare. In fact, close to one third (32%) of respondents to the household budget survey reported not using healthcare when needed, with half listing lack of affordability as the main reason for foregoing healthcare. In addition, the majority of those experiencing unmet healthcare needs are in the most socio-economically deprived segments of the population. For example, over half of respondents in the lowest asset index quintile reported unmet healthcare needs. It is important to note, therefore, that expenditure surveys ought to be combined with questions about unmet healthcare service needs.

### Limitations

Our study has several limitations. First, our findings only establish correlational rather than causal relationship. Additionally, survey bias associated with the recall period is a known limitation in similar studies and may have influenced the accuracy of reported data. Another constraint is the exclusion of Lower Shabelle from the SDHS survey due to security concerns, preventing the construction of a sub-national index for that region. Likewise, the SDHS survey had limited representation in Bay region, meaning the index for that area should be interpreted with caution. Lastly, the surveys did not include data on healthcare facility availability, which restricted our ability to analyse the direct relationship between healthcare infrastructure and progress toward UHC.

## Conclusions

In this paper, we assessed Somalia’s progress toward achieving SDG-3-related UHC targets. With an overall value of 33.5, our results place Somalia in last place in a list of 120 countries included in previous analyses. This situation, in part, reflects the broader systemic challenges facing the country, including insufficient funding, shortages of medical personnel, a fragmented healthcare infrastructure, and persistent political instability. Poverty and education attainment are among the main barriers preventing the country from further advancing on its path toward UHC. In addition, we found evidence of system failure, i.e., given the high cost of healthcare, Somalis simply does not seek healthcare services when needed. Finally, we also found that about a third of the population experiences unmet healthcare needs.

Against this background, the Somali government as well as the three regional authorities could take two approaches to advance its efforts toward improving UHC. First, authorities should build on existing small-scale community-provided healthcare service pilot projects. The Female Community Health Workers Program, consisting of 12 months of training, places female health workers in communities and provides basic medical supplies for basic medical treatment [16]. These healthcare workers conduct 5–7 home visits daily, each serving rural communities ranging from 600 to 1,000 people. Their responsibilities include delivering essential healthcare services, with a focus on maternal and child health, such as antenatal care, immunization, treatment of common childhood illnesses, and reproductive health services. Additionally, they track vital demographic events, including births, deaths, and population movements within communities [9, 16]. This approach has proven particularly effective in reaching rural and nomadic populations, who face the most significant challenges in accessing healthcare. Given the funding challenges that the country faces, a public-private partnership could ensure sustainability of the program. Furthermore, and in parallel, authorities should continue their efforts to enhance and strengthen the primary healthcare system, complemented by outreach programs. This strategy would enable the consistent delivery of affordable basic healthcare services, especially for urban populations [27]. Ultimately, given the findings of the analysis and the critical role of education and socioeconomic status in determining access to healthcare, these efforts should be integrated into a broader, comprehensive poverty reduction strategy.

## Electronic supplementary material

Below is the link to the electronic supplementary material.

Supplementary Material 1

## Data Availability

No datasets were generated or analysed during the current study.
