# Supplementary material for: Universal health coverage in fragile and conflict-affected States: insights from Somalia
Source: Int J Equity Health. 2025 May 7;24:125. doi: 10.1186/s12939-025-02486-3 (PMC12060528; doi:10.1186/s12939-025-02486-3)
Supplement: Supplementary file 1 — Supplementary Material 1 [file 12939_2025_2486_MOESM1_ESM.docx]

*Appendix 1. Details of the UHC index*

Following Wagstaff and Neelsen (2020), our UHC index is a geometric mean of two sets of indicators: service coverage (SC) and financial protection (FP). As discussed in the main text, these are equal to 100-CATA, where CATA is catastrophic healthcare expenditure:

𝑈𝐻𝐶 ≡ 𝑆𝐶 ^0.5^𝐹𝑃 ^0.5^ = 𝑆𝐶 ^0.5^ (100 − 𝐶𝐴𝑇𝐴) ^0.5^.

This approach creates an index that captures both UHC dimensions, allowing progress in one dimension to be traded off against progress in the other, reflecting the willingness among policy makers to often accept worse performance on one dimension in exchange for better performance on the other (Wagstaff and Neelsen, 2020).

The crucial step in creating the index is selecting the index elements. We were guided by previous studies by Wagstaff et al (2020), by Hogan et al (2018), and by six broad principles: (i) indicators should be delivered by health providers and should not capture downstream or upstream indicators; (ii) indicators should be as comprehensive as possible; (iii) indicators should reflect national priorities as much as international priorities based on SDG Agenda 2030; (iv) indicators should be based on nationally (and, in our case, sub-nationally) representative surveys; (v) each indicator should be able to be transformed into a coverage indicator; and (iv) indicators should be based on available data (Wagstaff and Neelsen, 2020).

References:

1. Wagstaff, Adam and Sven Neelsen (2020) “A comprehensive assessment of universal health coverage in 111 countries: a retrospective observational study”, Lancet Global Health, Volume 8, ISSUE 1, e39-e49, January 01, 2020.
2. Hogan DR, Stevens GA, Hosseinpoor AR, Boerma T. Monitoring universal health coverage within the Sustainable Development Goals: development and baseline data for an index of essential health services. The Lancet Global health 2018; 6(2): e152-e68.

*Appendix 2. Concentration index for equity of utilization*

We used the standard concentration index (CI) to assess the equity of utilization and the decomposition of CI to quantify the degree of equality in the utilization of health services and the extent to which our main variables of interest contributed to inequality of utilization (O’Donnell et al, 2007).

CI is defined with reference to the concentration curve. It is twice the area between the concentration curve and the line of equality (the 45-degree line). Concentration curves plot a specific health variable on the y-axis against the percentage distribution based on a wealth measure on the x-axis. Therefore, CI takes a value ranging from (-1 to 1) with negative values expressing pro-poor concentration and positive values expressing pro-rich concentration. Equation B presents the general model for CI:

$C=\frac{2}{\mu} {cov}_{w}(y_{i}, r_{i})$ (B)

where *C* is the CI, *y*_i_ is the measure of utilization of healthcare services, µ is the mean of *y*_i_, and *r_i_* is the rank distribution of individual *i* according to their wealth index.

The decomposition of the CI shows the contribution of the independent variables in the logit model to the distribution (inequality) of health services based on the wealth ranking of the population. It provides more detailed information and highlights potential areas for policy intervention. As equation C depicts, we used a probit model and its ‘partial effects’ (i.e., the effects of an individual independent variable, ceteris paribus) to conduct the decomposition analysis:

$E\left( y_{i} | x_{i} \right)=G \left( \sum_{k} \beta_{k}x_{k}^{i} \right)$ (C)

where G is the functional form for a nonlinear model. As proposed by van Doorslaer et al (2004), we restored the mechanics of the decomposition framework by replacing the βk parameters in the equation with βmk parameters, with βmk representing the partial effects of x (the determinants of y) in the linear approximation of the non-linear model expressed by Equation D:

$y_{i}= \sum_{k} \beta_{k}^{m}x_{k}^{i}+ \mu_{i}$ (D)

Accordingly, we conducted a decomposition of socio-economic related inequality affecting the healthcare utilization of our main variables of interest. For the purpose of this paper, we conducted decomposition analysis for the set of indicators exhibiting pro-rich inequity. Our choice of independent variables for use in the decomposition analysis was based on the availability of data in the SDHS. As such, the battery of independent variables captured enabling factors (e.g., educational attainment, wealth index, age) as well as community level factors (urbanicity and region of residence).

Educational attainment was captured by a continuous variable reporting number of years of schooling. The wealth index was the standard categorical variable with the five categories included in the SDHS (poorest, poor, average, rich, richest). Age was a continuous variable capturing the age (in years) of the respondent. Finally, two additional variables were used as proxies for the community level variables of urbanicity (a dummy variable that captured if the respondent lived in an urban, rural, or nomadic setting) and region.

References:

1. O’Donnell O, Van Doorslaer E, Wagstaff A, Lindelow M. Analyzing health equity using household survey data: a guide to techniques and their implementation. The World Bank; 2007.
2. van Doorslaer E, Koolman X, Jones AM. Explaining income-related inequalities in doctor utilization in Europe. Health Econ. 2004;13:629–47.

*Appendix 3. Catastrophic healthcare expenditure, its determinants, and Sartori model of those determinants*

***Catastrophic healthcare expenditure***

In order to derive catastrophic healthcare expenditure (CHE), we relied on the standard approach, i.e., estimating the share of households experiencing catastrophic healthcare expenditure. $N$ is the number of households and $E$ is a binary indicator that takes the value of 1 if out-of-pocket (oop) healthcare expenditure as a fraction of total healthcare expenditure ${(oop}_{i}/{exp}_{i})$ is greater than the respective threshold $z$, and 0 otherwise. The headcount can be thus expressed as follows:

$$Headcount=\frac{1}{N}\sum_{i=1}^{N} E_{i}$$

Total oop relied on both individual and household modules of the household survey. Data from the individual module included the following oop categories: expenditure on outpatient care, expenditure on inpatient care, expenditure on transport when seeking healthcare, and expenditure on overnight stays when seeking healthcare. We used data on pharmaceutical expenditure from the household module of the survey.

Finally, we used four different thresholds when calculating CHE: 10%, 15%, 20%, and 25% of total household consumption. Other CHE metrics (e.g., capacity to pay approach) have been used elsewhere to calculate catastrophic payments as a share of total expenditure minus spending on food (see Wagstaff, 2019). These measures have received some criticism recently; therefore, we did not use them in our analysis.

***Determinants of CHE***

Based on existing literature, we commenced our analysis using standard logit modelling, using the thresholds above to create four different CHE binary variables as the dependent variables in our models. In this context, if we assumed a linear model, the probability of incurring CHE could be analyzed by regressing the CHE variable (y_i_) on a vector of variables capturing household (x), community (u), and regional (z) characteristics according to the following equation:

$y_{i}^{*}=\alpha+\sum_{k} \beta_{k}x_{k,i}+\sum_{q} \delta_{q}u_{q,i}+ \sum_{p} \gamma_{p,i}z +\varepsilon_{i}$ , with i = 1,…N *(A)*

where α, β, γ_,_ δ are the parameters and ε_i_ is the error term.

Assuming that y_i_^*^ in equation (A) is a latent variable, the logit model is written as:

$$\left\{ \begin{aligned} 1 if y_{i}^{*}>0 \\ 0, otherwise \end{aligned} \right.$$

We used the following set of characteristics in our models.

Characteristic of the household: We used the following variables to capture household characteristics: (i) age of the household head; (ii) gender of the household head; (iii) a dummy variable capturing if the household head attended school or not; (iv) quintile of consumption to which the household belongs; and (v) variable capturing if the household is urban, rural, or nomadic.

Characteristics of the regions: We included a set of dummy variables capturing the characteristics of each region.

References:

1. Brown, S., Hole, A.R., Kilic, D., 2014. Out-of-pocket health care expenditure in Turkey: Analysis of the 2003–2008 Household Budget Surveys. Econ. Model. 41, 211–218.
2. Sartori, A.E., 2003. An Estimator for Some Binary-Outcome Selection Models Without Exclusion.
3. Kawabata K, Xu K, Carrin G. Preventing impoverishment through protection against catastrophic health expenditure. Bull World Health Organ 2002; 80: 612.

Wagstaff Adam (2019). Measuring catastrophic medical expenditures: Reflections on three issues. Health Economics. 2019;28(6):765–781.

1. Wagstaff A. Measuring catastrophic medical expenditures: Reflections on three issues. Health Economics. 2019;28(6):765–781

Appendix Table A1. UHC index and coverage of selected interventions (as %), national and subnational analysis

| UHC index | Share of the dimension in the UHC index | Dimensions | Share of the domain in UHC dimensions | Domain | Share of indicator in the respective domain | Indicator | Indicator definition |
| --- | --- | --- | --- | --- | --- | --- | --- |
|  | 50% | Service coverage | 25% | Prevention | 50% | 4 ante-natal care visits | Percentage of most recent birth in last five years with at least 4 antenatal care visits (women aged 15–49 at time of survey) |
|  |  |  |  |  | 50% | Full vaccination | Percentage of children aged 15–23 months who received (a) Bacillus Calmette-Guérin (BCG) against TB, (b) 3 doses of diphtheria-pertussis-tetanus (DPT)/Pentavalent, (c) 3 doses of polio (excluding polio given at birth), and (d) Measles/Measles-Mumps Rubella (MMR), either verified by vaccination card or by recall of respondent. |
|  |  |  | 75% | Treatment | 16.6% | Professionally-assisted childbirth | Percentage of most recent birth in last 5 years attended by any skilled health personnel (women age 15–49 at time of survey). Professional assistant includes doctor, nurse, midwife, and auxiliary midwife. |
|  |  |  |  |  | 16.6% | Treatment for acute respiratory infection (ARI) | Percentage of children under 5 years with cough and rapid breathing in the two weeks preceding the survey who had a consultation with a formal healthcare provider. |
|  |  |  |  |  | 16.6% | Treatment for diarrhea | Percentage of children under 5 years with diarrhea symptoms in the two weeks preceding the survey who had a consultation with a formal health care provider. |
|  |  |  |  |  | 50% | Inpatient care use in last 12 months (% of population age 18 years and older) | Percentage of population aged 18 years and older using inpatient care in the last 12 months. |
|  | 50% | Financial protection | 100% | Catastrophic expenses | 100% | Catastrophic expenditure at 10% | Percentage of population with out-of-pocket health expenses exceeding 10% of household consumption or income. |

Figure A2. Correlation between UHC index and poverty rate in Somalia, using CHE threshold of 10%

*Source:* SDHS 2020, SIHBS 2022, and author’s calculations. Correlation coefficient between UHC index and poverty rate is -0.

Appendix Table A2. Somalia: Decomposition analysis of the concentration index (CI) analysis for selected interventions

| **Medical assistance during delivery** | |  |  |  |  |
| --- | --- | --- | --- | --- | --- |
|  | Elasticity | CI | Contribution | percentage Contribution |  |
| Mother's age | -0.12 | 0.00 | 0.00 | 0.00 |  |
| Married | -0.19 | -0.02 | 0.00 | 0.01 |  |
| Attended school | 0.07 | 0.46 | 0.03 | 0.09 |  |
| Wealth index | 1.30 | 0.27 | 0.36 | 0.98 |  |
| Health insurance | 0.00 | 0.29 | 0.00 | 0.00 |  |
| Urban | -0.02 | -0.01 | 0.00 | 0.00 |  |
| Region | -0.41 | 0.01 | 0.00 | -0.01 |  |
|  |  |  |  |  |  |
| **Full childhood immunization** | |  |  |  |  |
|  | Elasticity | CI | Contribution | Percentage contribution | |
| Mother's age | 0.56 | 0.00 | 0.00 | 0.01 |  |
| Married | 0.12 | -0.02 | 0.00 | -0.02 |  |
| Attended school | 0.05 | 0.46 | 0.02 | 0.21 |  |
| Wealth index | 0.34 | 0.27 | 0.09 | 0.85 |  |
| Health insurance | 0.00 | 0.29 | 0.00 | 0.00 |  |
| Urban | -0.07 | -0.01 | 0.00 | 0.01 |  |
| Region | 0.90 | 0.01 | 0.01 | 0.06 |  |
|  |  |  |  |  |  |
| **Four ante natal care visits** | | |  |  |  |
|  | Elasticity | CI | Contribution | Percentage contribution | |
| Mother's age | 0.04 | 0.00 | 0.00 | 0.00 |  |
| Married | -0.01 | -0.02 | 0.00 | 0.00 |  |
| Attended school | 0.07 | 0.46 | 0.03 | 0.09 |  |
| Wealth index | 1.10 | 0.27 | 0.30 | 0.78 |  |
| Health insurance | 0.00 | 0.29 | 0.00 | 0.00 |  |
| Urban | -0.02 | -0.01 | 0.00 | 0.00 |  |
| Region | -1.57 | 0.01 | -0.01 | -0.03 |  |
|  |  |  |  |  |  |
| **Care seeking for diarrhea** | | |  |  |  |
|  | Elasticity | CI | Contribution | Percentage contribution | |
| Mother's age | -0.29 | 0.00 | 0.00 | 0.00 |  |
| Married | -0.18 | -0.02 | 0.00 | 0.02 |  |
| Attended school | 0.04 | 0.46 | 0.02 | 0.11 |  |
| Wealth index | 0.58 | 0.27 | 0.16 | 0.99 |  |
| Health insurance | |  |  |  |  |
| Urban | -0.01 | -0.01 | 0.00 | 0.00 |  |
| Region | -0.23 | 0.01 | 0.00 | -0.01 |  |
|  |  |  |  |  |  |
| **Care seeking for ARI** | | |  |  |  |
|  | Elasticity | CI | Contribution | Percentage contribution | |
| Mother's age | -0.38 | 0.00 | 0.00 | 0.00 |  |
| Married | -0.04 | -0.02 | 0.00 | 0.00 |  |
| Attended school | 0.05 | 0.46 | 0.02 | 0.10 |  |
| Wealth index | 0.83 | 0.27 | 0.23 | 1.09 |  |
| Health insurance | |  |  |  |  |
| Urban | -0.03 | -0.01 | 0.00 | 0.00 |  |
| Region | -0.50 | 0.01 | 0.00 | -0.02 |  |
|  |  |  |  |  |  |
| **Inpatient admissions** | | |  |  |  |
|  | Elasticity | CI | Contribution | Percentage contribution | |
| Age | -0.13 | -0.02 | 0.00 | 0.01 |  |
| Married | 0.15 | -0.06 | -0.01 | -0.04 |  |
| Attended school | -0.06 | 0.18 | -0.01 | -0.05 |  |
| Wealth index | 0.19 | 0.26 | 0.05 | 0.23 |  |
| Health insurance | |  |  |  |  |
| Urban | 0.39 | 0.23 | 0.09 | 0.43 |  |
| Region | 0.29 | -0.03 | -0.01 | -0.05 |  |

*Source:* SDHS 2020, SIHBS 2022, and author’s calculations.

Appendix Table A3. Logistic regression, determinants of CHE at 25% cut-off

| che_25 | Coef. | | St.Err. | t-value | | p-value | [95% Conf | | Interval] | Sig |
| --- | --- | --- | --- | --- | --- | --- | --- | --- | --- | --- |
| Age | 0.995 | | 0.004 | -1.19 | | 0.235 | 0.986 | | 1.003 |  |
| Female | 0.770 | | 0.336 | -0.60 | | 0.550 | 0.327 | | 1.812 |  |
| went_to_school | 2.048 | | 1.111 | 1.32 | | 0.186 | 0.708 | | 5.928 |  |
| 1b.region_n | 1.000 | | . | . | | . | . | | . |  |
| 2o.region_n | 1.000 | | . | . | | . | . | | . |  |
| 3o.region_n | 1.000 | | . | . | | . | . | | . |  |
| 4.region_n | 1.799 | | 2.075 | 0.51 | | 0.611 | 0.188 | | 17.253 |  |
| 5o.region_n | 1.000 | | . | . | | . | . | | . |  |
| 6.region_n | 1.013 | | 1.505 | 0.01 | | 0.993 | 0.055 | | 18.629 |  |
| 7o.region_n | 1.000 | | . | . | | . | . | | . |  |
| 8.region_n | 0.845 | | 1.118 | -0.13 | | 0.899 | 0.063 | | 11.296 |  |
| 9o.region_n | 1.000 | | . | . | | . | . | | . |  |
| 10o.region_n | 1.000 | | . | . | | . | . | | . |  |
| 11.region_n | 1.084 | | 1.288 | 0.07 | | 0.946 | 0.105 | | 11.131 |  |
| 12o.region_n | 1.000 | | . | . | | . | . | | . |  |
| 13.region_n | 3.803 | | 4.319 | 1.18 | | 0.240 | 0.410 | | 35.229 |  |
| 14o.region_n | 1.000 | | . | . | | . | . | | . |  |
| 15.region_n | 3.496 | | 4.004 | 1.09 | | 0.275 | 0.370 | | 33.003 |  |
| 16.region_n | 1.419 | | 1.758 | 0.28 | | 0.778 | 0.125 | | 16.085 |  |
| 17.region_n | 2.471 | | 3.184 | 0.70 | | 0.483 | 0.198 | | 30.873 |  |
| 1b.ea_type_n | 1.000 | | . | . | | . | . | | . |  |
| 2.ea_type_n | 0.634 | | 0.351 | -0.82 | | 0.410 | 0.214 | | 1.876 |  |
| 3.ea_type_n | 0.973 | | 0.600 | -0.04 | | 0.964 | 0.290 | | 3.261 |  |
| 1b.quintile | 1.000 | | . | . | | . | . | | . |  |
| 2.quintile | 1.316 | | 0.860 | 0.42 | | 0.674 | 0.366 | | 4.739 |  |
| 3.quintile | 0.347 | | 0.308 | -1.19 | | 0.233 | 0.061 | | 1.977 |  |
| 4.quintile | 0.523 | | 0.496 | -0.68 | | 0.494 | 0.081 | | 3.357 |  |
| 5o.quintile | 1.000 | | . | . | | . | . | | . |  |
| Constant | 0.009 | | 0.010 | -4.50 | | 0.000 | 0.001 | | 0.072 | *** |
|  | | | | | | | | | | |
| Mean dependent var | | 0.010 | | | SD dependent var | | | 0.099 | |  |
| Pseudo r-squared | | 0.076 | | | Number of obs | | | 3028.000 | |  |
| Chi-square | | 20.722 | | | Prob > chi2 | | | 0.189 | |  |
| Akaike crit. (AIC) | | 109309.491 | | | Bayesian crit. (BIC) | | | 109411.757 | |  |
|  | | | | | | | | | | |
| **** p<0.01, ** p<0.05, * p<0.1* | | | | | | | | | |  |
